# A machine learning-based predictive model for predicting early neurological deterioration in lenticulostriate atheromatous disease-related infarction

**DOI:** 10.3389/fnins.2024.1496810

**Published:** 2024-12-11

**Authors:** Zhuangzhuang Jiang, Dongjuan Xu, Hongfei Li, Xiaolan Wu, Yuan Fang, Chen Lou

**Affiliations:** Department of Neurology, Dongyang People’s Hospital, Affiliated to Wenzhou Medical University, Dongyang, China

**Keywords:** early neurological deterioration, branch atheromatous disease, lenticulostriate artery, ischemic stroke, machine learning

## Abstract

**Background and aim:**

This study aimed to develop a predictive model for early neurological deterioration (END) in branch atheromatous disease (BAD) affecting the lenticulostriate artery (LSA) territory using machine learning. Additionally, it aimed to explore the underlying mechanisms of END occurrence in this context.

**Methods:**

We conducted a retrospective analysis of consecutive ischemic stroke patients with BAD in the LSA territory admitted to Dongyang People’s Hospital from January 1, 2018, to September 30, 2023. Significant predictors were identified using LASSO regression, and nine machine learning algorithms were employed to construct models. The logistic regression model demonstrated superior performance and was selected for further analysis.

**Results:**

A total of 380 patients were included, with 268 in the training set and 112 in the validation set. Logistic regression identified stroke history, systolic pressure, conglomerated beads sign, middle cerebral artery (MCA) shape, and parent artery stenosis as significant predictors of END. The developed nomogram exhibited good discriminative ability and calibration. Additionally, the decision curve analysis indicated the practical clinical utility of the nomogram.

**Conclusion:**

The novel nomogram incorporating systolic pressure, stroke history, conglomerated beads sign, parent artery stenosis, and MCA shape provides a practical tool for assessing the risk of early neurological deterioration in BAD affecting the LSA territory. This model enhances clinical decision-making and personalized treatment strategies.

## Introduction

Branch atheromatous disease (BAD) is a common cause of progressive ischemic stroke (Deguchi and Takahashi, 2023; Petrone et al., 2016). Among the penetrating arteries, the lenticulostriate artery (LSA) has larger ramification zones and a more intricate course (Marinkovic et al., 1985). Consequently, lesions in the LSA are more likely to induce early neurological deterioration (END), characterized by motor deficits (Liu H. et al., 2023).

Currently, antithrombotic agents such as argatroban and tirofiban have been proven effective in treating progressive stroke, including cases associated with BAD (Zhang X. et al., 2024; Du et al., 2022; Zhang et al., 2022). However, in clinical practice, concerns persist about the routine use of these novel antithrombotic agents in the context of pre-progression ischemic stroke due to the potential risk of hemorrhage. Therefore, identifying and closely monitoring high-risk individuals for END is beneficial for timely and precise medication administration.

Currently, numerous studies are investigating the risk factors for END in BAD. However, some studies focus solely on single subcortical infarction (Nam et al., 2021b; Nam et al., 2021a; Jang et al., 2020), disregarding the distinct pathogenic mechanisms between lacunar infarction caused by lipohyalinotic degeneration and subcortical infarction associated with BAD (Kwan et al., 2011; Sun et al., 2017), potentially introducing selection bias. Additionally, some studies indiscriminately investigate all penetrating arteries (Wu et al., 2020; Oji et al., 2018), overlooking the influence of parent artery morphology on penetrating artery disease (Ha et al., 2022; Jeong et al., 2015; Liu et al., 2024). Furthermore, there is currently a lack of predictive models to systematically forecast END caused by BAD in the LSA territory. The objective of this article is to establish a model for predicting END in BAD-related ischemic stroke in the LSA territory through machine learning. Furthermore, the study aims to explore the underlying mechanisms of END occurrence in the LSA territory.

## Materials and methods

### Patients

In this retrospective observational study, we exclusively examined consecutive ischemic stroke patients with BAD in the LSA territory at Dongyang People’s Hospital between January 1, 2018, and September 30, 2023. Ethical approval was obtained from the Ethics Committee of Dongyang People’s Hospital, and the study strictly adhered to the principles outlined in the Declaration of Helsinki. Personal information was concealed throughout data extraction and analysis to ensure patient privacy protection. Patients were included based on the following criteria: (1) admission within 48 h of symptom onset; (2) meeting the diagnostic criteria for stroke related to BAD in the LSA territory, defined as follows: diffusion-weighted imaging (DWI) indicating that the infarct extended over three or more slices within the territory supplied by the lenticulostriate artery (Deguchi et al., 2013), encompassing the basal ganglia, corona radiata, and internal capsule; (3) complete cranial and cervical computed tomography angiography (CTA). The imaging evaluation was completed 48 h after admission. Exclusion criteria consisted of: (1) significant (> 50%) stenosis of the cerebral middle artery (MCA), internal carotid artery, or common carotid artery ipsilateral to the infarct; (2) potential sources of cardioembolism, such as atrial fibrillation, cardiomyopathy, and valvular heart disease; (3) other potential causes of ischemic stroke, such as dissection, patent foramen ovale, antiphospholipid syndrome, and Moyamoya disease; (4) missing data.

### Data collection

We collected a comprehensive set of baseline characteristics, including demographic information, vascular risk factors, pre-admission and post-admission medication use, blood pressure at admission, and laboratory test results on admission or the following day. Hypertension was defined as the previous use of antihypertensive medication, systolic blood pressure > 140 mm Hg, or diastolic blood pressure > 90 mm Hg at discharge (Whitworth, 2003). Diabetes mellitus was defined as the previous use of glucose-lowering medication or hemoglobin A1c ≥6.5% (Rayburn, 1997). Hyperlipidemia was defined as the previous use of lipid-lowering medication, fasting low-density lipoprotein cholesterol >160 mg/dL, or fasting total cholesterol >240 mg/dL (Expert Panel on Detection, Evaluation, and Treatment of High Blood Cholesterol in Adults, 2001). Ischemic heart disease (IHD) was considered present if there was a clear medical history or if the condition was definitively diagnosed at discharge. A prior history of stroke was defined as a history of transient ischemic attack or ischemic stroke (Wang et al., 2011). Cigarette smoking was defined as a history of smoking at least one cigarette per day for 6 months or more (Tong et al., 2016). Alcohol consumption was defined as consuming 15 g or more alcoholic drinks per day in the previous year (Lemarchand et al., 2015). The medication history of antiplatelet and statin use referred to regular drug usage before admission, irrespective of drug type and dosage. Recombinant tissue plasminogen activator (RTPA) was administered following the standard protocol: a dose of 0.9 mg/kg, not exceeding 90 mg total, infused over 60 min, with an initial bolus of 10% of the dose delivered within the first minute (Powers et al., 2018). All patients received antiplatelet therapy upon admission, including monotherapy with either aspirin or clopidogrel, or dual antiplatelet therapy with aspirin combined with clopidogrel, regardless of dosage and loading status. All patients were administered statins orally upon admission, irrespective of dosage and statin type.

### Neuroimaging protocol and analysis

**Figure 1:**
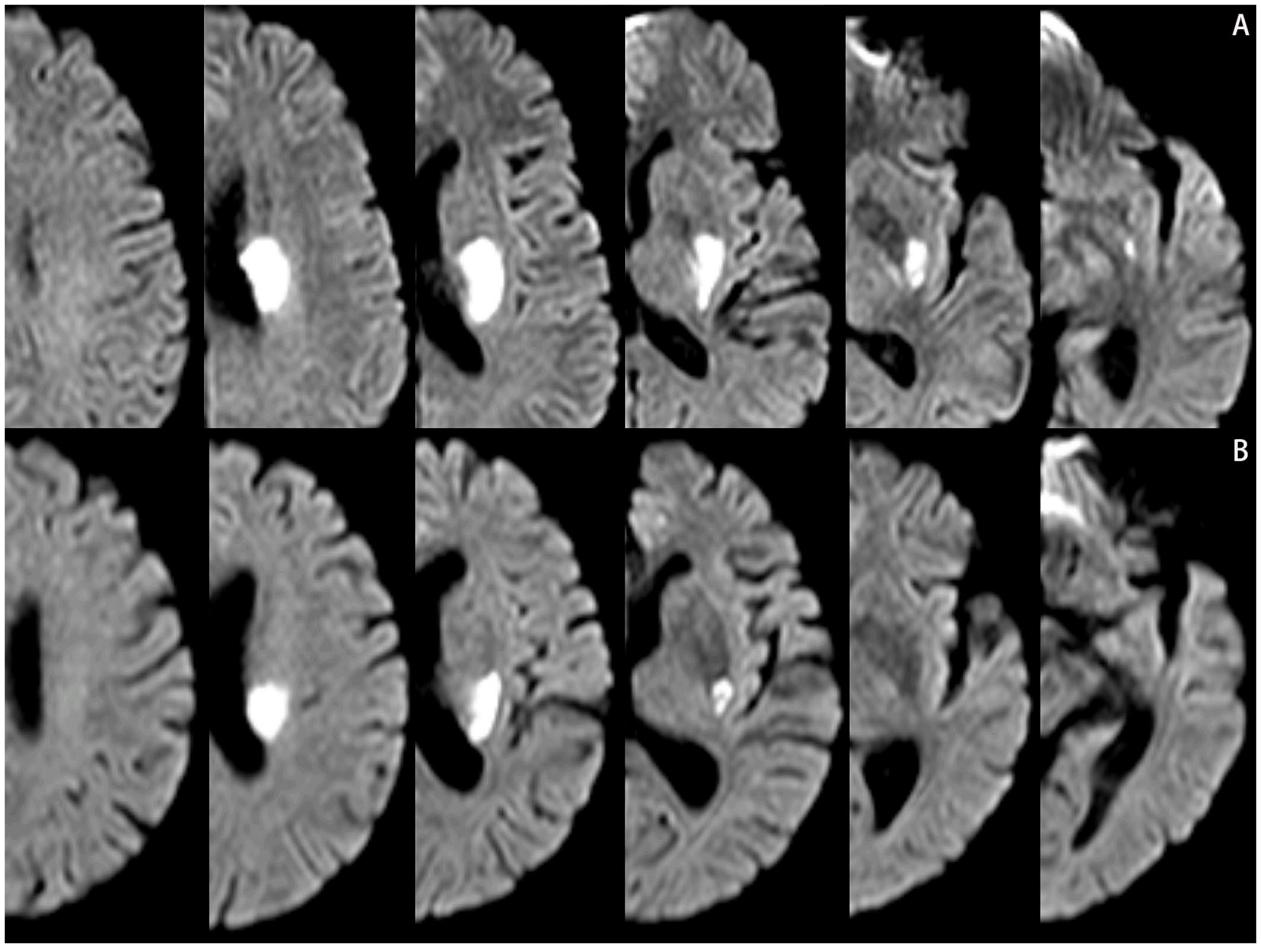
Lesions location on the diffusion-weighted MRI. **(A)** Proximal single subcortical infarction. **(B)** Distal single subcortical infarction.

**Figure 2:**
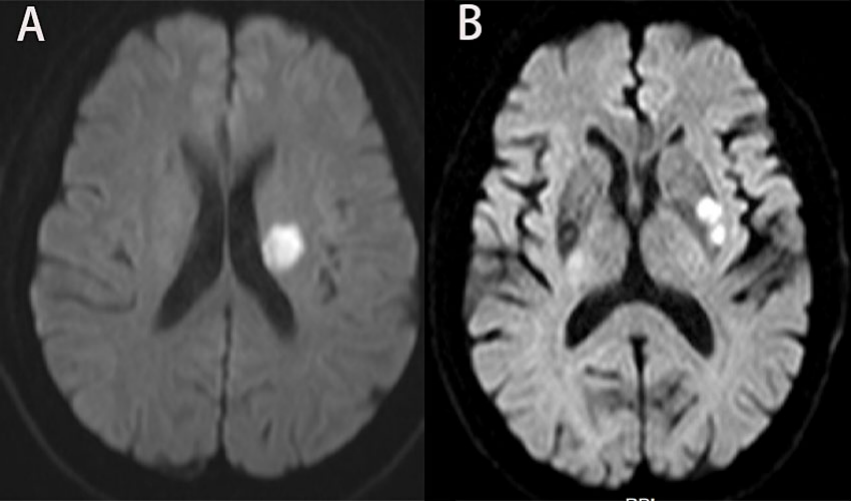
Lesions pattern on the diffusion-weighted MRI. **(A)** Single infarcts are observed in the left corona radiata. These infarcts were designated as oval shape without conglomerated beads sign. **(B)** Grouped infarcts in the left basal ganglia, defined as conglomerated beads shape, are observed.

**Figure 3:**
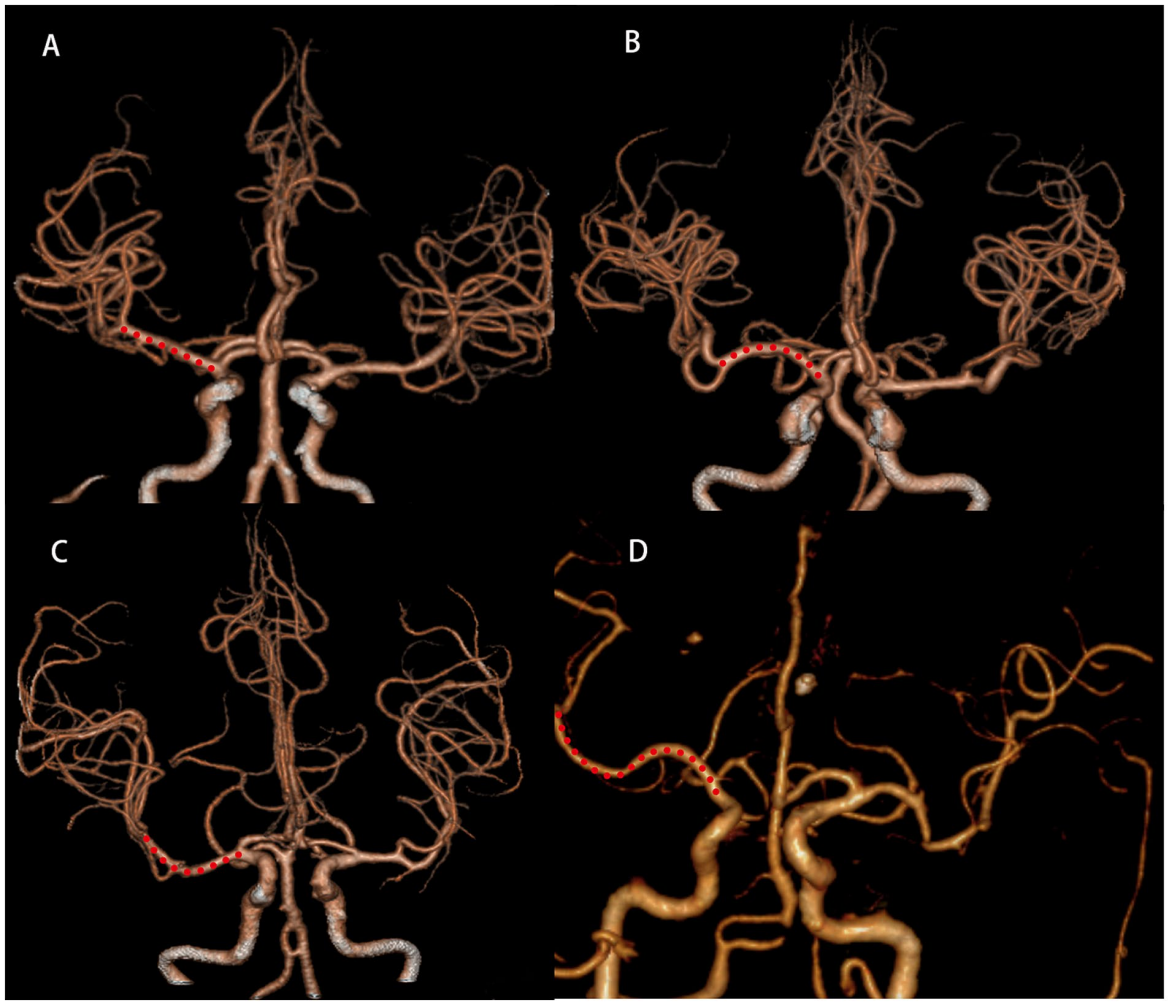
Shape of MCA from three-dimensional reconstructed image of CTA. Straight **(A)**, inverted U-shaped **(B)**, U-shaped **(C)** and S-shaped MCAs **(D)**. MCA, middle cerebral artery; CTA, computed tomography angiography.

The scanner parameters for DWI were as follows: repetition time, 7,500 ms; echo time, 84 ms; matrix size, 128 × 128; two b values, 0 and 1,000 s/mm^2^; slice thickness, 5 mm; and inter-slice gap, 2 mm. The scanner parameters for CTA included 100 kVp, 200 mAs, and 0.625 mm axial slice thickness. After intravenous injection of 100 mL of non-ionic contrast material, serial axial thin sections were obtained from the aortic arch to the vertex. Patients were categorized into PSSI (proximal single subcortical infarction) and DSSI (distal single subcortical infarction) groups according to the involvement of the lowest portion of the basal ganglia (Zhang et al., 2014) (Figure 1). PSSI was considered as an infarction extending to the basal surface of the MCA. Lesions on the axial DWI plane were classified into two groups based on shape: those presenting without and those presenting with a conglomerated beads sign (Ryu et al., 2012) (Figure 2). The maximum length and width of the largest infarction area on an axial view were measured. The number of axial image slices showing cerebral infarction was counted. The infarct volume of the selected slice with the largest lesion observed on DWI was measured using the ABC/2 method: 0.5 × diameter of the length × diameter of the width × (0.5 × number of DWI slices with acute infarction) (Zhang et al., 2014). Parent artery stenosis was defined as 0 to 50% narrowing in the M1 segment of the MCA (Jeong et al., 2015). Asymptomatic stenosis was defined as a stenosis of ≥50% of the intracranial large artery that was not associated with the infarct (Yang et al., 2023a). The number of asymptomatic intracranial stenoses in each participant was counted. From the three-dimensional reconstructed image of CTA, the shape of the MCA was measured between the anterior cerebral artery (ACA)-MCA bifurcation and the M2 bifurcation in the anterior–posterior direction. MCA shape was classified into four groups: (1) straight; (2) inverted U-shaped; (3) U-shaped; and (4) S-shaped MCA (Ha et al., 2022; Kim et al., 2015) (Figure 3). Neuroimaging was independently assessed by two experienced neurologists, with disputes resolved through group discussions.

### Definition of END

All patients were admitted within 48 h after the onset of symptoms, and MRI examinations were completed within 48 h after admission. The NIHSS score was checked daily until hospital discharge. END was immediately reported after its development to the staff on duty or to neurology residents. END was defined as an increase of ≥2 points in the total NIHSS score compared to the NIHSS score at admission during the first week of admission (Liu Y. et al., 2023; Wu et al., 2020). The END should not be attributed to other conditions such as symptomatic hemorrhagic transformation, infection, electrolyte disturbance, medication side effects, or other significant medical comorbidities (Jeong et al., 2015). Considering the heterogeneity of NIHSS score assessment in medical records, our third-party assessment team, blinded to the study group assignment and treatment, conducted a central assessment of END based on medical records. If the assessment results were inconsistent with the previous assessment, the third assessor intervened in the assessment.

### Statistical analysis

Continuous variables were assessed for normality using the Kolmogorov–Smirnov test and presented as either medians with interquartile ranges (IQRs) or means with standard deviations (SD), depending on their distribution. Categorical variables were expressed as numbers and percentages. Comparisons between two groups of continuous variables were made using the independent t-test for normally distributed data and the Mann–Whitney U-test for non-normally distributed data. For categorical variables, differences between the groups were analyzed using Fisher’s exact test or the Chi-square (χ^2^) test, as appropriate.

LASSO regression analysis was employed to identify the most significant features. Nine machine learning algorithms were applied: Logistic Regression (LR) used the glm function from the stats package to model binary outcomes, followed by examining coefficients and *p*-values using the summary function. Support Vector Machine (SVM) parameters were optimized using the tune.svm function from the e1071 package through cross-validation. Gradient Boosting Machine (GBM) from the gbm package iteratively built decision tree ensembles to enhance prediction accuracy. Artificial Neural Networks (ANN) constructed customizable networks using the nnet package to capture complex data relationships. Tree Bag (TG) employed random forest to create ensembles with bootstrapped samples and feature randomness, preventing overfitting. Partial Least Squares (PLS) through pls extracted components maximizing predictor-outcome covariance. Neural Networks configured via neuralnet adapted for regression and classification tasks. Bayesian Classifiers using naiveBayes assumed predictor independence for categorical data analysis. Random Forests powered by randomForest built robust decision tree ensembles suited for high-dimensional datasets, resistant to overfitting.

The performance of models in both the training and validation sets was evaluated using the area under the curve (AUC). The model achieving the highest AUC in the validation set was identified as the optimal model. Identified risk factors were used to construct a nomogram, with calibration assessed via the Hosmer-Lemeshow goodness-of-fit test and calibration plots with 500 bootstrap resamples. Decision Curve Analysis (DCA) was conducted in both sets to evaluate the clinical utility of the nomogram. All statistical tests were two-tailed with a significance level of *p* < 0.05. Analyses were performed using R version 4.0.4 and SPSS version 26.

## Results

### Baseline patient characteristics

**Figure 4:**
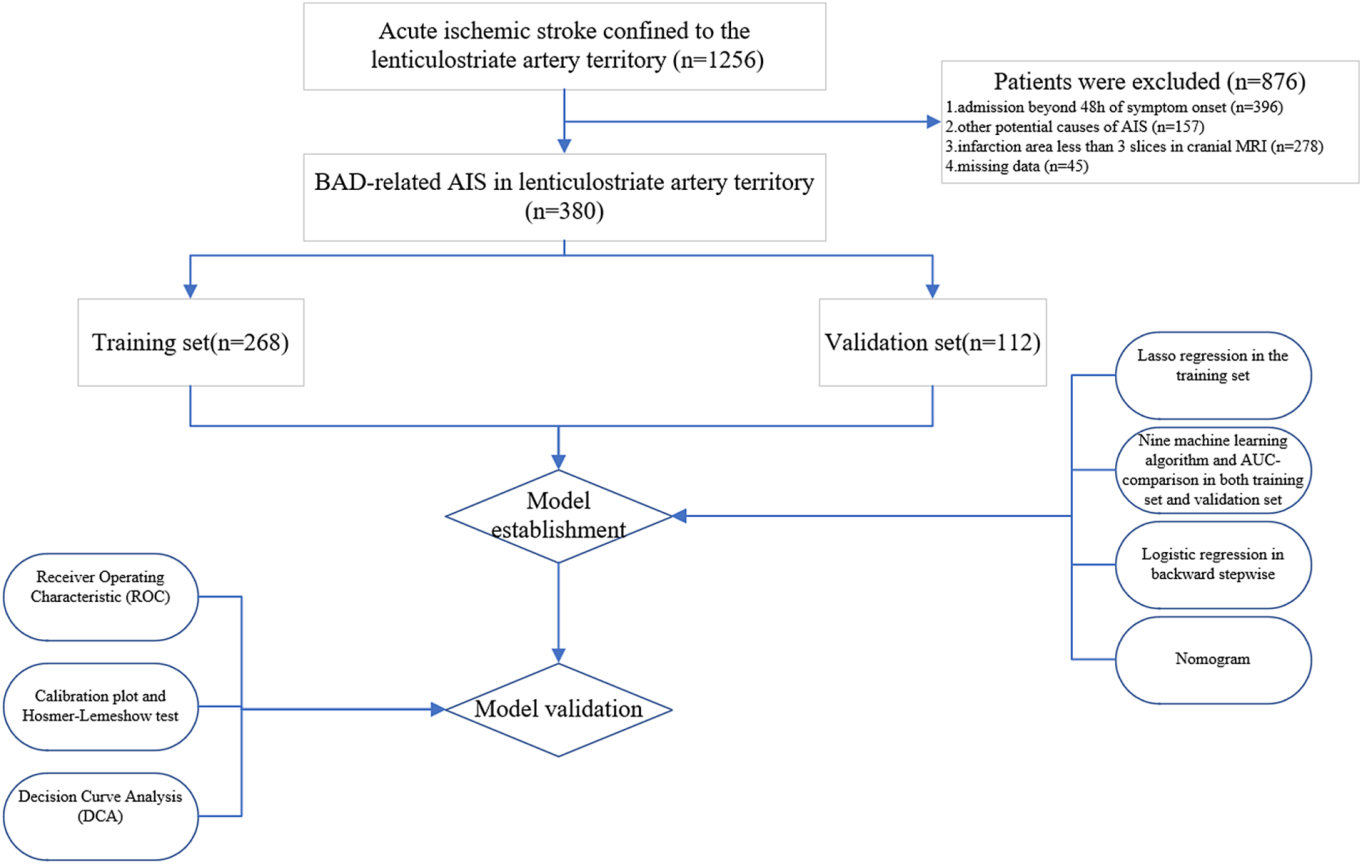
Flow diagram of study design. AIS, acute ischemic stroke; MRI, magnetic resonance imaging; BAD, branch atheromatous disease; AUC, area under the curve.

A total of 380 patients were included in this study. Among them, 268 individuals were assigned to the training set, and the remaining 112 individuals formed the validation set in a 7:3 ratio (Figure 4). Table 1 displays the baseline characteristics of the patients in both sets. The percentages of patients with END were 23.1% in the training set and 16.1% in the validation set. All variables were found to be balanced between the two groups, with *p*-values exceeding 0.05, indicating no significant differences.

**Table 1:** Baseline characteristics of BAD-related AIS in lenticulostriate artery territory: Training set vs. Validation set.

Variables	All patients (*n* = 380)	Training set (*n* = 268)	Validation set (*n* = 112)	*p-*value
Demographic data				
Age (years), median (IQR)	66.00 (55.00, 74.00)	65.00 (55.00, 74.00)	66.50 (55.75, 74.25)	0.974
Sex, male, *n* (%)	211 (55.5)	145 (54.1)	66 (58.9)	0.388
Weight (kg), mean (SD)	61.79 (12.03)	61.59 (11.60)	62.26 (13.04)	0.622
Height (cm), median (IQR)	162.00 (157.00, 169.0)	162.00 (157.00, 169.00)	163.00 (158.00, 168.25)	0.526
BMI, mean (SD)	23.37 (3.93)	23.37 (3.90)	23.36 (4.02)	0.973
Vascular risk factors, *n* (%)				
Hypertension	292 (76.8)	210 (78.4)	82 (73.2)	0.278
Diabetes mellitus	70 (18.4)	44 (16.4)	26 (23.2)	0.119
Hyperlipidemia	76 (20.0)	55 (20.5)	21 (18.8)	0.694
Ischemic heart disease	51 (13.4)	39 (14.6)	12 (10.7)	0.317
Previous stroke	33 (8.7)	21 (7.8)	12 (10.7)	0364
Smoke- current or previous	128 (33.7)	88 (32.8)	40 (35.7)	0.588
Drinking	93 (24.5)	63 (23.5)	30 (26.8)	0.498
Prior medication, *n* (%)				
Antiplatelet	18 (4.7)	16 (6.0)	2 (1.8)	0.080
Statin	17 (4.5)	14 (5.2)	3 (2.7)	0.274
Post-admission medication, *n* (%)				
IVT	52 (13.7)	41 (15.3)	11 (9.8)	0.157
Antiplatelet				0.361
Aspirin alone	120 (31.6)	88 (32.8)	32 (28.6)	
Clopidogrel alone	88 (23.2)	65 (24.3)	23 (20.5)	
DAPT	172 (45.3)	115 (42.9)	57 (50.9)	
Baseline data				
NIHSS (scores), median (IQR)	3.00 (2.00, 5.00)	3.00 (2.00, 5.00)	3.00 (2.00, 4.25)	0.882
SBP (mmHg), mean (SD)	161.66 (22.16)	162.02 (21.89)	160.79 (22.88)	0.624
DBP (mmHg), median (IQR)	89.00 (79.00, 100.00)	89.00(80.00, 101.00)	87.50 (79.00, 99.00)	0.175
END, *n* (%)	80 (21.1)	62 (23.1)	18 (16.1)	0.124
Laboratory data, median (IQR)				
WBC (*10^9^/L), median (IQR)	6.92 (5.69, 8.27)	7.05 (5.78, 8.50)	6.59 (5.38, 8.04)	0.074
Neutrophil (*10^9^/L), median (IQR)	4.50 (3.46, 5.61)	4.54 (3.58, 5.62)	4.20 (3.28, 5.61)	0.234
Lymphocyte (*10^9^/L), median (IQR)	1.71 (1.27, 2.21)	1.71 (1.29, 2.23)	1.70 (1.24, 2.09)	0.369
NLR, median (IQR)	2.60 (1.80, 3.70)	2.50 (1.87, 3.70)	2.85 (1.80, 3.80)	0.607
RBC (*10^12^/L), mean (SD)	4.65 (0.55)	4.68 (0.54)	4.57 (0.56)	0.064
Hemoglobin (g/L), mean (SD)	142.88 (17.75)	143.93 (17.52)	140.37 (18.13)	0.074
Platelet (*10^9^/L), median (IQR)	210.50 (174.75, 248.00)	207.50 (176.00, 244.75)	212.00 (170.50, 251.00)	0.806
MPV (fL), median (IQR)	9.50 (8.90, 10.30)	9.50 (8.90, 10.30)	9.55 (8.88, 10.10)	0.324
Serum uric acid (μmol/L), median (IQR)	286.00 (239.00, 347.00)	288.00 (239.00, 348.25)	284.50 (241.75, 339.25)	0.619
Serum creatinine (μmol/L), median (IQR)	61.00 (52.00, 72.25)	60.00 (52.00, 73.00)	63.50 (52.00, 72.00)	0.281
Sua/Scr, median (IQR)	4.70 (3.90, 5.70)	4.70 (4.00, 5.80)	4.40 (3.77, 5.40)	0.060
Blood sugar (mmol/L), median (IQR)	5.14 (4.70, 5.87)	5.14 (4.70, 5.81)	5.20 (4.71, 6.32)	0.557
HbA1c (%), median (IQR)	5.70 (5.40, 6.10)	5.70 (5.40, 6.10)	5.80 (5.38, 6.28)	0.275
TG (mmol/L), median (IQR)	1.31 (0.92, 1.81)	1.32 (0.92, 1.83)	1.30 (0.91, 1.79)	0.998
LDL (mmol/L), mean (SD)	2.85 (0.86)	2.85 (0.90)	2.85 (0.77)	0.963
HDL (mmol/L), median (IQR)	1.09 (0.93, 1.30)	1.08 (0.93, 1.29)	1.12 (0.93, 1.31)	0.929
TC (mmol/L), median (IQR)	4.50 (3.96, 5.15)	4.50 (3.94, 5.15)	4.53 (3.97, 5.13)	0.798
Neuroimaging data				
Onset to initial MRI (h), median (IQR)	45.00 (33.00, 61.00)	45.50 (33.00, 58.25)	49.00 (34.75, 70.00)	0.148
PSSI, n (%)	219 (57.6)	154 (57.5)	65 (58.0%)	0.918
CBS, n (%)	103 (27.1)	73 (27.2)	30 (26.8)	0.928
Layers (slice), median (IQR)	4.00 (3.00, 4.00)	4.00 (3.00, 4.00)	4.00 (3.00, 4.00)	0.889
Length (mm), median (IQR)	17.00 (14.00, 23.33)	17.00 (14.00, 23.02)	17.65 (13.00, 24.00)	0.949
Width (mm), median (IQR)	9.15 (7.92, 13.00)	9.00 (8.00, 13.00)	10.00 (7.55, 13.93)	0.403
Volume (cm^3^), median (IQR)	2.80 (1.80, 5.00)	2.80 (1.78, 4.73)	2.80 (1.80, 5.62)	0.697
PAS, n (%)	81 (21.3)	54 (20.1)	27 (24.1)	0.390
Asymptomatic stenosis, mean (SD)	0.23 (0.59)	0.24 (0.61)	0.21 (0.56)	0.756
MCA shape, *n* (%)				0.712
Straight	107 (28.2)	77 (28.7)	30 (26.8)	
U-shape	105 (27.6)	71 (26.5)	34 (30.4)	
Inverted U-shape	54 (14.2)	41 (15.3)	13 (11.6)	
S-shape	114 (30.0)	79 (29.5)	35 (31.2)	

BAD, branch atheromatous disease; AIS, acute ischemic stroke; IVT, intravenous thrombolysis; DAPT, dual antiplatelet therapy; NIHSS, National Institutes of Health Stroke Scale; SBP, systolic blood pressure; DBP, diastolic blood pressure; END, early neurological deterioration; WBC, white blood cell; NLR, neutrophil-to-lymphocyte; ratio; RBC, red blood cell; MPV, mean platelet volume; Sua/Scr, serum uric acid-to-serum creatinine ratio; HbA1c, glycosylated hemoglobin; TG, triglycerides; LDL, low-density lipoprotein cholesterol; HDL, high-density lipoprotein cholesterol; TC, total cholesterol; MRI, magnetic resonance imaging; PSSI, proximal single subcortical infarction; CBS, conglomerated beads shape; PAS, parental arterial disease; MCA, middle cerebral artery.

### Machine learning model evaluation

**Figure 5:**
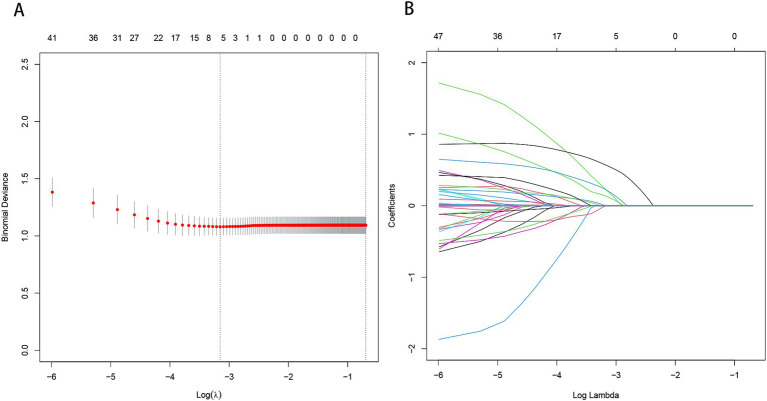
Predictor selection using the LASSO regression analysis with five-fold cross-validation. **(A)** The binomial deviance curve with error bar is plotted against log (*λ*), where λ is the tuning parameter. The dotted vertical lines are drawn at the optimal values by minimum criteria and the one standard error of the minimum criteria (1se criteria). **(B)** A coefficient profile plot was created against the log (lambda) sequence. In this study, predictor’s selection was according to the minimum criteria, where 5 nonzero coefficients were selected. LASSO, least absolute shrinkage and selection operator.

**Figure 6:**
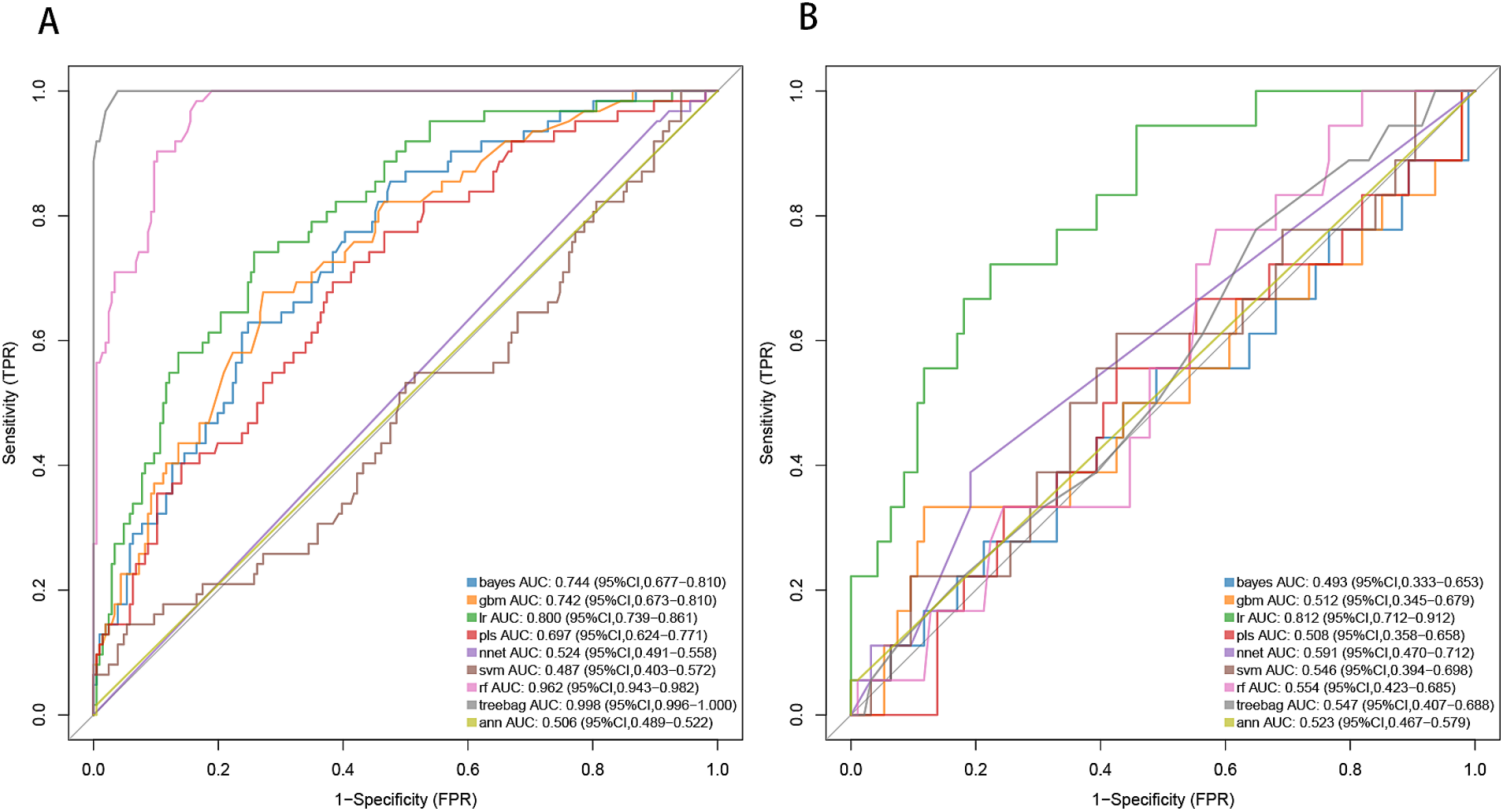
ROC curve analysis of machine learning algorithms for prediction of END in BAD-related ischemic stroke in LSA territory. END, early neurological deterioration; BAD, branch atheromatous disease; LSA, lenticulostriate artery. **(A)** the training set, **(B)** the validation set.

By applying LASSO regression filtration in the training set, we identified systolic pressure, stroke history, conglomerated beads sign, parent artery stenosis, and MCA shape as significant predictors (Figure 5). We employed nine machine learning algorithms (LR, SVM, GBM, ANN, Treebag, PLS, NNET, Bayes, RF) to construct models using these five predictors. The performance of the models was assessed using ROC curves for both the training and validation sets (Figure 6). In the training set, the treebag model achieved the highest AUC of 0.998 (95% CI, 0.996–1.000). However, in the validation set, the logistic regression model exhibited the highest AUC of 0.812 (95% CI, 0.712–0.912) compared to the other models (*p* < 0.05, DeLong test). Consequently, the logistic regression model was selected for further analysis due to its superior performance in the validation set.

### Predictive model development

The predictors, including stroke history, systolic pressure, conglomerated beads sign, MCA shape, and parent artery stenosis, were entered into the multivariable logistic regression analysis using the backward stepwise method. All variables remained in the final model: stroke history (OR, 2.472; 95% CI, 0.933–6.548; *p* = 0.069), systolic pressure (OR, 1.01; 95% CI, 0.996–1.025; *p* = 0.154), conglomerated beads sign (OR, 2.617; 95% CI, 1.387–4.940; *p* = 0.003), MCA shape (straight MCA OR, 1.368; 95% CI, 0.419–4.47; *p* = 0.604; U-shape MCA OR, 2.63; 95% CI, 0.856–8.078; *p* = 0.091; S-shape MCA OR, 2.902; 95% CI, 0.968–8.704; *p* = 0.057; compared to inverted U-shaped), and parent artery stenosis (OR, 2.025; 95% CI, 1.01–4.058; *p* = 0.047) (Table 2).

**Table 2:** Logistic regression analysis for predicting END in BAD-related AIS in LSA territory.

Variable	OR	95%CI	*P*-value
Conglomerated beads shape	2.617	(1.387, 4.940)	0.003
Parent artery stenosis	2.025	(1.010, 4.058)	0.047
Systolic pressure	1.010	(0.996, 1.025)	0.154
Stroke history	2.472	(0.933, 6.548)	0.069
Inverted-U shaped MCA	Reference		
Straight MCA	1.368	(0.419, 4.470)	0.604
U-shaped MCA	2.630	(0.856, 8.078)	0.091
S-shaped MCA	2.902	(0.968, 8.704)	0.057

END, early neurological deterioration; BAD, branch atheromatous disease; AIS, acute ischemic stroke; LSA, lenticulostriate artery; MCA, middle cerebral artery.

### Development and evaluation of nomogram

**Figure 7:**
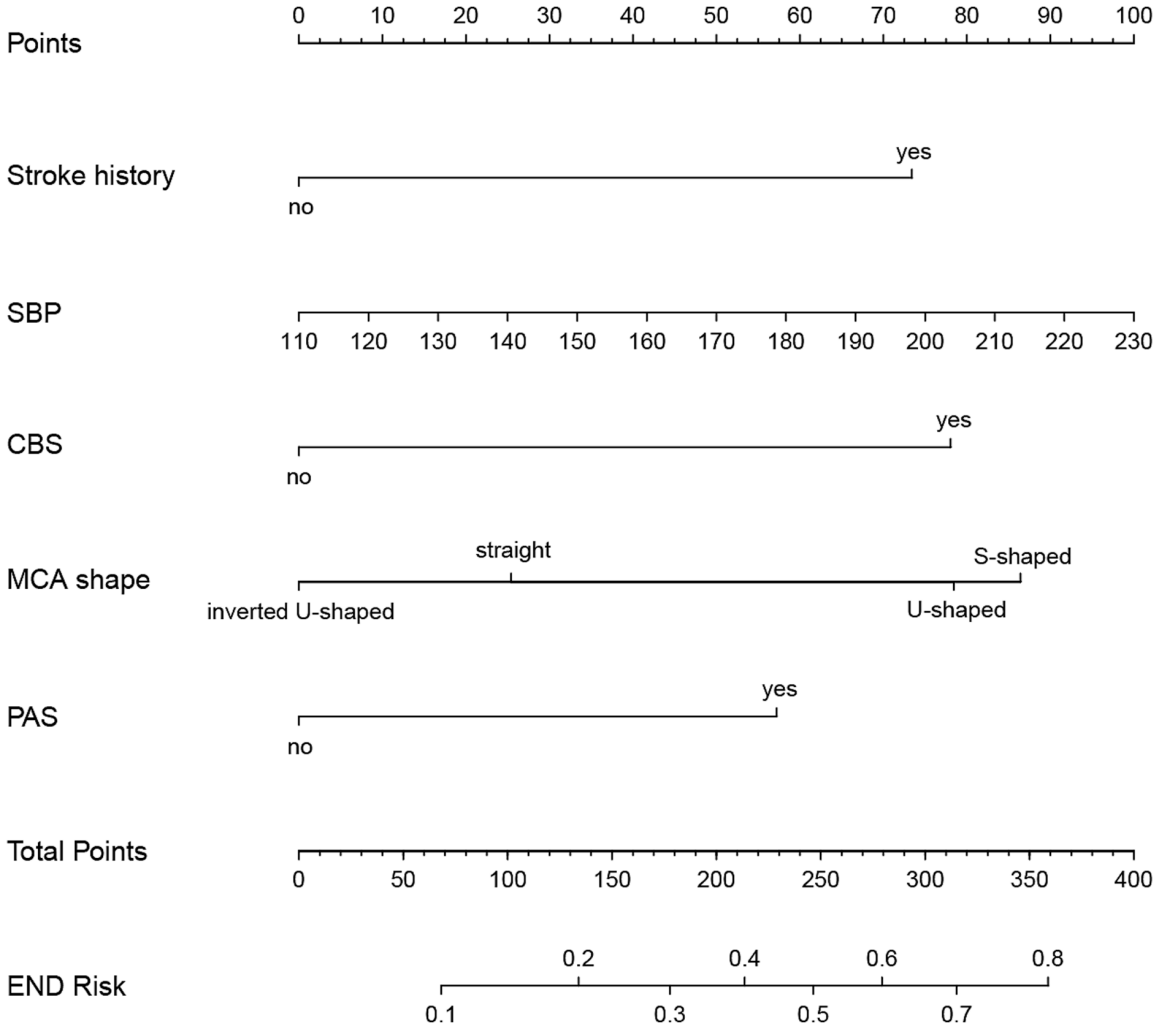
Nomogram for predicting END in BAD-related ischemic stroke in LSA territory. The nomogram consists of five predictors, each of which is given a preliminary score (0–100). The total score is obtained by summing all the preliminary score of each of the three predictors. SBP, systolic blood pressure; CBS, conglomerated beads shape; MCA, middle cerebral artery; PAS, parental arterial stenosis; LSA, lenticulostriate artery.

**Figure 8:**
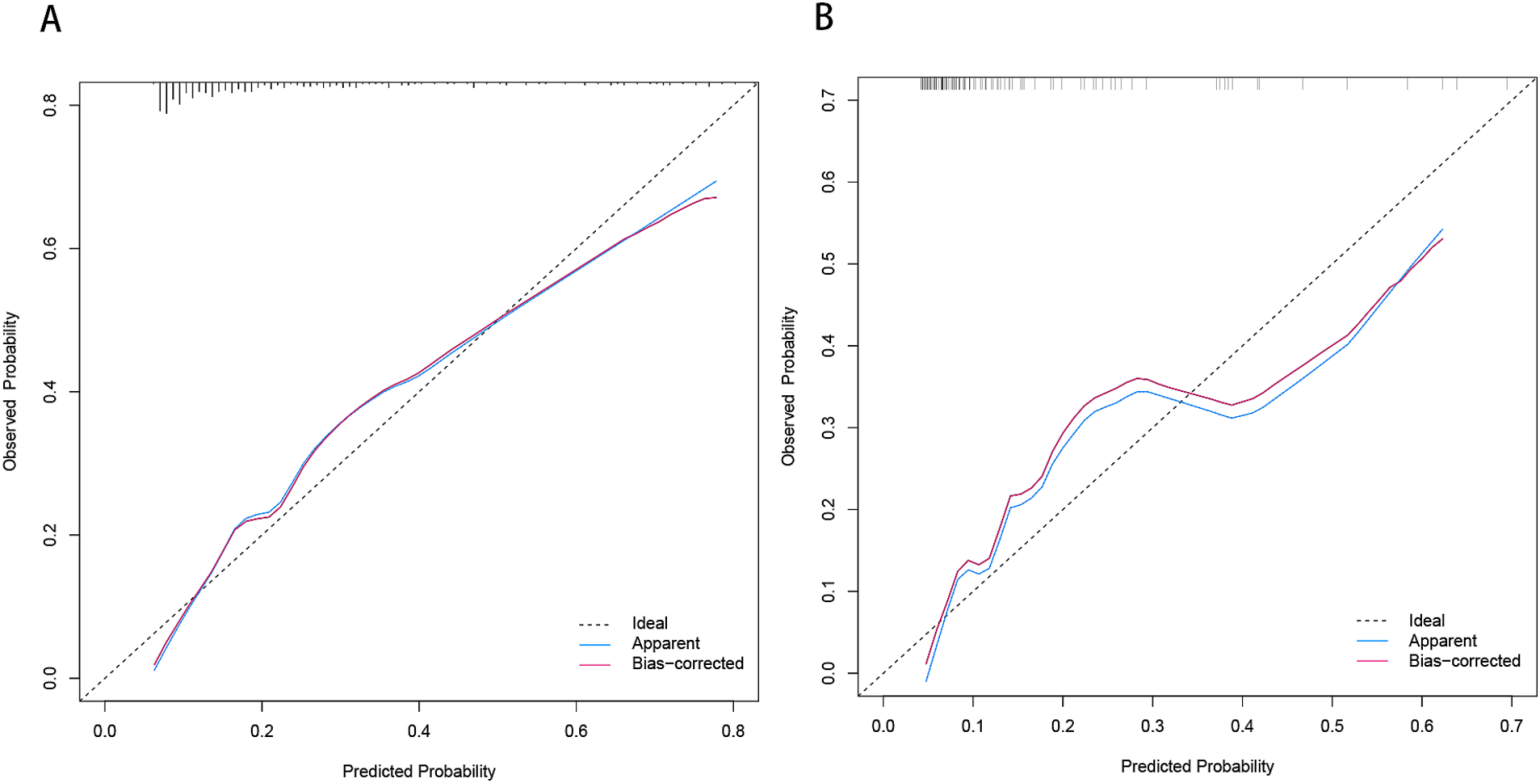
Calibration plot for predicting END in BAD-related ischemic stroke in LSA territory in the training set **(A)** and the validation set **(B)**. END, early neurological deterioration; BAD, branch atheromatous disease; LSA, lenticulostriate artery.

**Figure 9:**
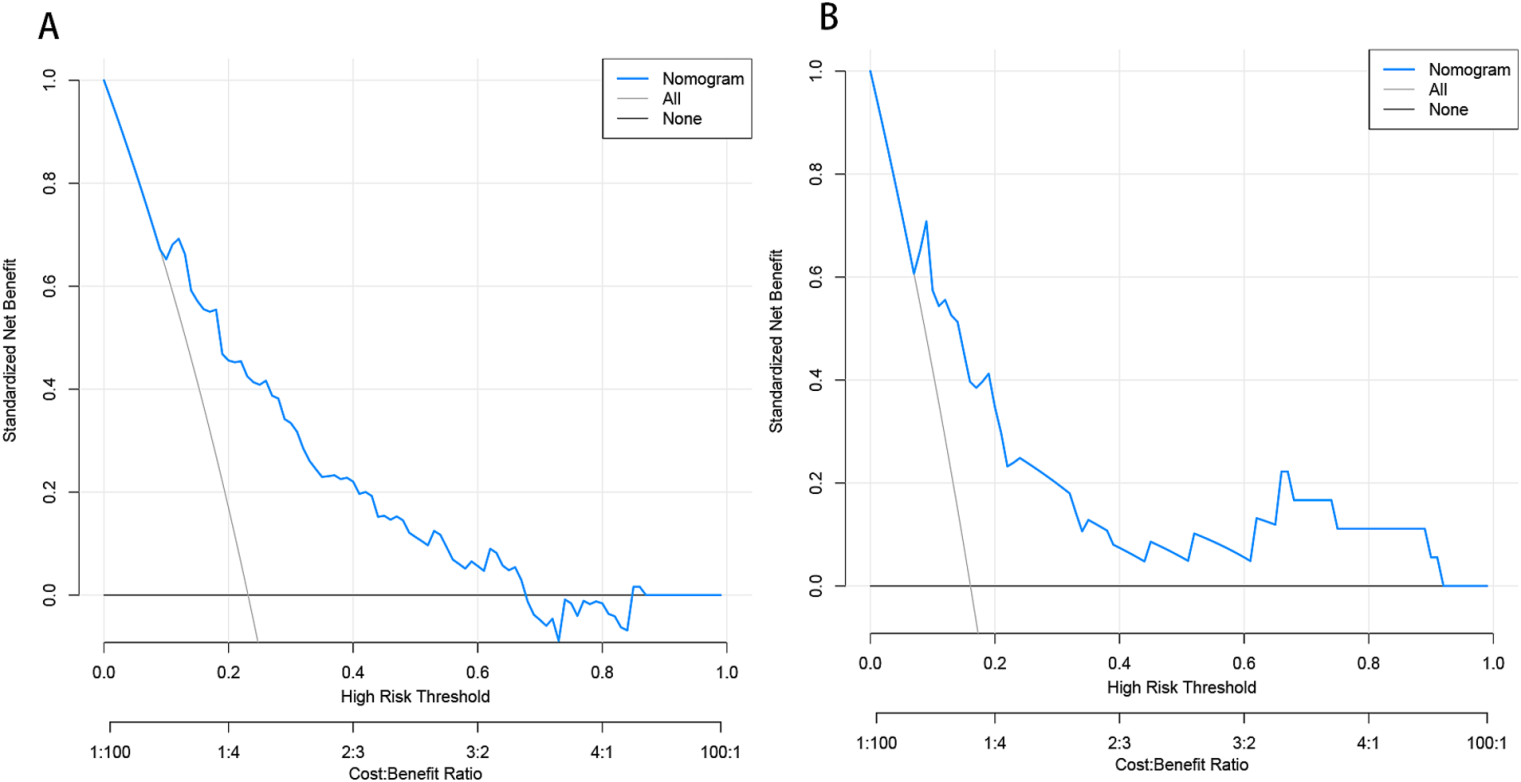
Decision curve analysis (DCA) of the nomogram predicting END in BAD-related ischemic stroke in LSA territory in the training set **(A)** and the validation set **(B)**. The x-axis demonstrates the threshold probability. The y-axis indicates the net benefit. The black line displays all patients are negative and have no treatment, the net benefit is zero. The gray line means all patients will develop END. The blue line indicates the net benefit of the nomogram. END, early neurological deterioration; BAD, branch atheromatous disease; LSA, lenticulostriate artery.

Based on the results from logistic regression analysis, we constructed a nomogram incorporating significant predictors (Figure 7). The discriminative ability of the nomogram was assessed using AUC, demonstrating moderate predictive power in both the training set (AUC, 0.800; 95% CI, 0.739–0.861) and the validation set (AUC, 0.812; 95% CI, 0.712–0.912) (Figure 6). Additionally, the goodness-of-fit of the nomogram was evaluated using the Hosmer-Lemeshow test, revealing good agreement between predicted and observed probabilities in both the training set (*p* = 0.640) and the validation set (*p* = 0.736). Calibration plots for both sets showed excellent alignment between predicted END probabilities and actual observations (Figure 8). To assess clinical utility, decision curve analysis (DCA) was conducted, indicating threshold probabilities ranging from 11 to 67% in the training set and 9 to 92% in the validation set (Figure 9).

## Discussion

In this study, we comprehensively incorporated various parameters to construct a model, including cerebrovascular disease risk factors, medications, laboratory tests, and imaging characteristics of cerebral vessels and infarct lesions. We utilized machine learning algorithms to construct predictive models. However, some algorithms demonstrated low AUC values in both the training and validation sets, indicating that they may not be well-suited to the dataset. Others, such as the tree bagging algorithm, performed well on the training set but showed lower AUC in the validation set, likely due to overfitting caused by an insufficient training sample size. In contrast, logistic regression showed strong and consistent performance across both the training and validation sets. We attribute this to two key factors. First, logistic regression is a well-established and reliable method in medical statistics, known for its robustness across diverse data types. Second, our training set adhered to the 10 EPV (events per variable) rule (Peduzzi et al., 1996), ensuring at least 10 positive outcomes per predictor variable, a critical factor for the stability and reliability of logistic regression. Finally, we apply logistical regression to develop and validate a novel nomogram for predicting END in BAD of the LSA territory. The final model comprises factors such as stroke history, systolic pressure, conglomerated beads sign, MCA shape, and parent artery stenosis. These risk factors are routinely collected in clinical practice, thereby enhancing the model’s practicality and applicability in clinical settings. Decision curve analysis (DCA) in the training set demonstrated that when an individual’s threshold probability ranges from 11 to 67%, this model offers greater net benefit compared to treat-all or treat-none strategies. In clinical practice, patients within this range require closer monitoring to ensure early detection of END. Further studies exploring whether early administration of argatroban and tirofiban in high-risk patients can reduce the incidence of END would be valuable.

We observed a positive correlation between systolic blood pressure at admission and the occurrence of END, consistent with prior study (Park et al., 2020). This association may stem from the interplay between blood pressure levels and the inflammatory response triggered by cerebral infarction (Di Napoli and Papa, 2006). Ischemic stroke initiates a robust inflammatory cascade in the brain, leading to neuroinflammation (Anrather and Iadecola, 2016). Hypertension has been proven to be associated with neuroinflammatory responses and atherosclerosis mediated by neurotransmitters (Di Napoli and Papa, 2005). We speculate the systolic blood pressure measured at the onset of acute cerebral infarction could indirectly indicate the severity of neuroinflammation, potentially causing cerebral edema and enlargement of the infarcted area, thereby exacerbating neurological deficits (Vila et al., 2000). In our study, we included parameters such as neutrophils, lymphocytes, and the neutrophil-lymphocyte ratio, but no correlation with END was found through univariate logistic regression across all samples. Further investigation is warranted to explore the relationship between other neuroinflammatory markers such as CRP, interleukins and END.

Previous studies have shown that in stroke patients undergoing thrombolysis or those with severe intracranial arterial stenosis or occlusion, stroke history increases the risk of stroke progression (Li N. et al., 2024; Li et al., 2020). In this study, we also observed an association between prior stroke and END in BAD-related stroke in LSA territory, which may explain why individuals with a history of stroke are at higher risk of poor long-term outcomes following recurrent stroke (Patti et al., 2019; Hsu et al., 2020). Patients with previous strokes often exhibit compromised collateral circulation (Ding et al., 2022; Leker et al., 2019), potentially leading to ischemic penumbra progression to core infarcts due to insufficient collateral perfusion (Kawano et al., 2016; Rusanen et al., 2015), exacerbating neurological deficits. However, our study also included the variable of moderate to severe asymptomatic intracranial stenosis, but found no association with END by univariate logistic regression across all samples. We speculate that mild asymptomatic intracranial stenosis could similarly affect collateral circulation in stroke.

Our study demonstrated that in ischemic stroke related to BAD, the morphology rather than the size of the infarct lesion correlates with END. Univariate regression analysis of the full sample showed that CBS remained significantly associated with END (*p* < 0.01), whereas all parameters related to infarct size showed no association with END. Previously, the conglomerated beads sign has been associated with stroke progression in patients with penetrating artery disease (Ryu et al., 2012; Yang et al., 2023b). Conglomerated bead-like lesions often indicate pathology at the trunk of perforating arteries or occlusion of perforating artery branches by parent artery plaques, resulting in downstream multifocal blockages and the appearance of multiple scattered adjacent lesions (Yang et al., 2023b; Yu and Tan, 2015). We speculate the mechanism of END is related to subsequent lesion coalescence leading to further enlargement of the stroke area. This phenomenon bears resemblance to the island sign or satellite sign observed during early hematoma expansion in patients with cerebral hemorrhage (Lv et al., 2021; Li et al., 2017). For stroke patients exhibiting the conglomerated beads sign, follow-up head MRI can help confirm this hypothesis. We did not find any correlation between the size of brain infarcts on admission DWI and early END. We believe that the initial infarct area size correlates primarily with the baseline NIHSS score, whereas the expansion of the infarct area is linked to END (Terasawa et al., 2008; Yamada et al., 2004).

BAD can cause cerebral infarction through two main mechanisms: occlusion at the origin of the penetrating artery due to an atherosclerotic plaque at the junction of the parent artery, or the presence of a microatheroma in the proximal segment of the penetrating artery (Pan et al., 2023; Petrone et al., 2016). The presence of atherosclerotic plaques in the parent vessel has been linked to progressive stroke (Wang et al., 2019). In this study, we observed that the morphology of the MCA was associated with the incidence of END. Specifically, a tortuous MCA is more prone to END. Previous researches have established a positive correlation between the tortuosity of the basilar and internal carotid arteries and plaque formation (Deng et al., 2021; Ren et al., 2023). Consequently, we hypothesize that S-shaped MCAs, being the most tortuous among the four MCA shapes, similarly increase plaque formation, potentially obstructing the origin of the penetrating artery and thereby elevating the risk of progressive stroke. Furthermore, we found that U-shaped MCAs were more likely to be associated with END compared to inverted U-shaped MCAs. We posit that U-shaped MCAs tend to develop plaques more frequently on their superior aspects, likely due to slower blood flow along the inner curve that facilitates lipid deposition (Kim et al., 2015). Anatomically, the LSA branches from the superior aspect of the MCA (Yan et al., 2023; Jiang et al., 2020), increasing the likelihood of plaques obstructing perforating arteries in U-shaped MCAs.

Consistent with previous studies, we found that ischemic stroke patients with MCA stenosis were at a higher risk of experiencing END (Zhang J. et al., 2024; Xie et al., 2021). Large artery atherosclerotic stroke with over 50% stenosis carries a higher risk of developing END compared to other ischemic stroke subtypes due to persistent perfusion deficits in the infarct area (Park et al., 2020). In our study, parent arteries with mild stenosis are also more likely to be associated with END compared to those without stenosis. This is because mild stenosis often indicates underlying atherosclerotic plaque (Nah et al., 2010; Men et al., 2024), thereby increasing the risk of obstruction at the openings of the perforating arteries.

Based on our findings, we speculate that plaque in the parent artery plays a significant role in the development of early neurological deterioration in lenticulostriate atheromatous disease-related infarction. In Chinese ischemic stroke sub classification (CISS) (Chen et al., 2012), occlusion of penetrating artery origins due to parent artery plaques is categorized as large artery atherosclerotic stroke, whereas pathology affecting the penetrating artery itself is classified as penetrating artery disease-related stroke. The potential role of parent artery plaques in progressive cerebral infarction indirectly supports the rationale behind the CISS classification.

To the best of our knowledge, this is the first clinical predictive model for END in lenticulostriate atheromatous disease-related infarction. Unlike previous studies that have focused on single subcortical infarctions (Yang et al., 2023a; Luo et al., 2024), we specifically target BAD-related subcortical infarctions, which are associated with higher disability and progression rates (Li S. et al., 2024), thus enhancing the clinical applicability of our model. Given the close relationship between BAD and parent artery (Ha et al., 2022; Huang et al., 2024), we incorporated the morphological features of the MCA and the degree of MCA stenosis into our model. This allows the model parameters to be interpreted in the context of the underlying pathophysiological mechanisms of END. However, this study has some limitations. Firstly, this retrospective study was conducted at a single center, potentially introducing biases and limiting the statistical robustness of the findings. Secondly, LASSO regression was applied for feature selection, which may overlook non-linear relationships and multicollinearity among variables. Future research could focus on developing models using different variable selection methods and comparing their reliability to evaluate the robustness of each approach. Thirdly, our study did not incorporate inflammatory markers such as CRP, which limits investigation into the influence of inflammatory response on the progression of cerebral infarction. Lastly, our study did not include high-resolution MRI to analyze plaque locations and the morphology of lenticulostriate arteries. However, high-resolution MRI is predominantly used in research settings and has not seen widespread adoption in clinical practice. Therefore, our model retains strong practical applicability in clinical settings.

## Conclusion

In conclusion, the newly developed nomogram incorporating systolic pressure, stroke history, conglomerated beads sign, parent artery stenosis, and MCA shape provides a predictive tool for assessing the risk of early neurological deterioration in branch atheromatous disease affecting the lenticulostriate artery territory.

## Data Availability

The raw data supporting the conclusions of this article will be made available by the authors, without undue reservation.
